# Impact of supplementary private health insurance on stomach cancer care in Korea: a cross-sectional study

**DOI:** 10.1186/1472-6963-9-133

**Published:** 2009-07-31

**Authors:** Dong Wook Shin, Kee-Taig Jung, Sung Kim, Jae-Moon Bae, Young-Woo Kim, Keun Won Ryu, Jun Ho Lee, Jae-Hyung Noh, Tae-Sung Sohn, Young Ho Yun

**Affiliations:** 1 National Cancer Control Research Institute & Hospital, National Cancer Center, Goyang, Gyeonggi, Korea; 2 Department of Health Services Management, Kyung Hee University, Seoul, Korea; 3 Department of Surgery, Samsung Medical Center, Sungkyunkwan University, Seoul, Korea

## Abstract

**Background:**

Korea achieved universal health insurance coverage in only 12 years; however, insufficient government funding has resulted in high out-of-pocket payments and, in turn, a demand for supplementary private health insurance (PHI). Supplementary PHI provides a fixed amount of benefits in the event of critical illness (e.g., cancer or stroke), surgery, or hospitalization. In this study, we tried to identify factors that influence the decision to purchase supplementary PHI and investigate the impacts of PHI on various aspects of cancer care.

**Methods:**

In a cross-sectional study of 391 patients with gastric cancer, we collected data on demographic and clinical variables, coverage by PHI at the time of diagnosis, and patients' cancer care experiences from surgery databases and patient questionnaires. Two separate multivariate logistic regression models were used 1) to determine whether various sociodemographic and clinical variables influence the purchase of supplementary PHI, and 2) to determine if there is a difference in various outcome measures between individuals with and without PHI.

**Results:**

We studied 187 subjects (49.6%) who were covered under PHI at the time of diagnosis. Subjects who purchased PHI tended to be younger (aOR = 5.01, 95% C.I. = 2.05 – 12.24), and more educated (aOR = 2.67, 95% C.I. = 1.04 – 6.86). Supplementary PHI coverage was significantly associated with financial independence (aOR = 2.07, 95% CI = 1.19 – 3.61), but not with other aspects of cancer care, such as access to healthcare, quality of care, communication and patient autonomy.

**Conclusion:**

Our findings demonstrate that supplementary PHI neither serves as a safety net for vulnerable patients nor improves cancer care experience, except for maintaining the financial independence of beneficiaries.

## Background

Many developing countries, spurred by rapid economic growth, are faced with increasing public aspirations for better and affordable health care. For example, when healthcare topped the list of concerns in China [1], the Chinese government released a draft of healthcare reform plans on Oct 14, 2008. These ambitious plans aim to provide safe, effective, convenient, and affordable basic care for all citizens by 2020 [2]. When attempting to achieve universal coverage, the most important question is how to finance the soaring health care costs. Despite increased spending, coverage by the Chinese government has decreased and out-of-pocket payments have risen, implying that insufficient funds are available [3,4]. In the meantime, the demand for private health insurance (PHI) has become intense and the market, particularly with regards to supplementary insurance, has dramatically expanded [5].

South Korea has experienced similar expedited economic development under an authoritarian political regime and has achieved universal coverage through the National Health Insurance (NHI) program in only 12 years. Thus, South Korea has been the subject of substantial interest for many developing countries, particularly those with low and middle incomes [6]. The Korean government began by introducing mandatory social health insurance for industrial workers in large corporations in 1977, then incrementally extended this program to cover self-employed citizens and eventually the entire population in 1989 [7]. On the other hand, the Korean government has been unable to control health care costs, and the Korean NHI is currently experiencing a financial deficit [8,9]. As the government puts a higher priority on extending the population coverage, the NHI has maintained a policy of 'low contributions, limited benefits, and high co-payments'.

The contribution rate in Korea was relatively low as of 2006, with an average of 4.48% of wage income, and the health insurance benefit package included diagnostic tests, drugs and medical materials, treatments and surgery, rehabilitation, and hospitalization. However, many services, especially expensive ones, were not covered by the NHI package. Furthermore, the co-payment rate was uniformly 20% for inpatient care, without a ceiling, and 35% to 50% for hospital outpatient care [6]. These rates lead to substantial out-of-pocket payments in Korea, accounting for 37% of the total health care costs in 2004 [10] and more for serious illnesses (e.g., approximately 50% for cancer) [11]. Limited coverage and excessive co-payments have resulted in a relatively low satisfaction rate (i.e., 47.0%) with the NHI [12] and rapid growth of the PHI market [13,14]. As the government did not allow for indemnity plans, most existing PHI plans were disease-specific products that provide a fixed amount of benefits in the event of critical illness (e.g., cancer or stroke), surgery, or hospitalization [14].

Recently, there has been intense debate over the government's encouragement of PHI in Korea. This controversy increased when the Korean government proposed a plan to develop the medical sector as an industry in 2003. Supporters of the plan argue that the private sector is driven by the need to attract clients, and is therefore in a position to enhance the efficiency and quality of health care, increase patient satisfaction, and relieve the financial burden caused by NHI. However, critics of this plan argue that the potential benefits of PHI have not yet been demonstrated, and the private sector may inadvertently contribute to higher NHI expenditures by spurring patient demand. Critics are also concerned that PHI could result in inequities that would further deepen the social disharmony among Koreans and create a sense of incongruity [6,15]. Thus far, this debate has been waged on theoretical ideas, rather than on concrete evidence.

Although a limited number of studies have examined the role of PHI on cancer care in the United States [16,17], the unique U.S. healthcare system and different socioeconomic background make it difficult to apply the findings to developing countries. Thus, we sought to identify factors that influence the decision to purchase supplementary PHI in Korea, and investigate the impacts of PHI on various aspects of cancer care, including access to healthcare, quality of care, communication, patient autonomy, and financial and social independence.

## Methods

### Study design

We performed a cross-sectional study in 2004 to examine various aspects of cancer care among gastric cancer patients. Until recently, gastric cancer was the most commonly diagnosed malignancy in Korea, accounting for 20% of new cancer cases in 2002 [18].

Patients were selected from two independent institution-based stomach surgery databases at the National Cancer Center and the Samsung Medical Center in Korea. Both databases have very similar data structures that include clinical information, such as age at diagnosis, disease stage, tumor progress, type of surgery (including extent of lymph node dissection), history of cancer therapy, and recurrence. Patients who were diagnosed with stage I to stage III stomach cancer between 2001 and 2002, and remained disease-free at the time of survey, were regarded eligible for participation in this study. Patients were excluded from the study if they had a prior history of other cancers.

Potentially eligible patients were invited to participate in the study by telephone, and those who agreed to participate were mailed the questionnaire, consent forms, and a postage-paid return envelope. Subjects who did not return the questionnaire within a month received a reminder card and were telephoned by a research staff member, who explained the purpose of the study and participation requirements. Subjects who remained interested were asked to sign an informed consent form, and complete and return the questionnaire. Subjects who chose not to participate were asked to provide reasons. The original study design and recruitment procedures have been previously described elsewhere [19]. The study was approved by the Institutional Review Boards of both medical centers.

### Measurements

We used a questionnaire to collect information on sociodemographic and clinical characteristics, including various aspects of cancer care. Sociodemographic variables included age, sex, marital status, education level, household income, residential area, religious status, and employment status before and after diagnosis. Clinical data included variables such as comorbidities, smoking and drinking habits (i.e., before and after diagnosis). Subjects were asked if they were covered by PHI at the time of diagnosis, and how helpful they found the PHI.

To determine the impacts of PHI, cancer care was assessed from various perspectives. 'Access to health care' and 'quality of care' were assessed objectively and subjectively. 'Stage at diagnosis' was used as an objective measure to assess timely access to health care [16], and 'problems receiving surgery after diagnosis' was assessed subjectively using a patient questionnaire. 'Guideline compliance' was used as an objective measure of the quality of care. Patients were considered to have been treated according to Korean Gastric Cancer Society (KGCS) guidelines [20] if they were diagnosed with stage I disease and received surgery (regardless of chemotherapy) or if they were diagnosed with stage II or III disease and received adjuvant chemotherapy (with or without radiotherapy). We did not consider the concept of over-treatment, as it was not relevant to our research questions. 'Overall satisfaction with care' was used as a subjective measure of the quality of care. Patient autonomy was assessed by asking subjects to rate 'the degree of their own involvement in decision making' and 'the extent that their treatment plans reflected their own opinions.' 'Payment of treatment costs by oneself' (i.e., not relying on financial assistance from a family member or friend) and 'job maintenance after cancer treatment' were used to assess financial and social independence, respectively. Items included in the questionnaire are described in detail in the Additional file 1.

### Statistical analysis

We used a univariate and standard multivariate logistic regression model to determine whether various sociodemographic and clinical variables influenced the purchase of supplementary PHI in Korea. Results are shown as odds ratios (OR) and adjusted odds ratios (aOR). A separate multivariate logistic regression model was used to assess differences in various outcome measures between persons with PHI and without PHI. Estimates were adjusted for possible confounding variables, such as age at diagnosis, educational status, religion, income, residential area, and employment status at the time of diagnosis. All statistical tests were two-sided, and *P *< 0.05 was considered statistically significant.

## Results

### Study participants

Hospital records yielded 855 patients who had undergone curative surgery for stomach cancer between 2001 and 2002, and we were able to contact 774 patient households by telephone. However, 83 patients had died and 97 refused to participate in the study because of time constraints, inability to communicate verbally or in writing (i.e., no one was available to help them), or the study was regarded as an inconvenience or a violation of privacy.

Of the remaining 594 patients, 165 did not return the questionnaire. Among the remaining 429 patients, 6 were excluded for not completing the questionnaire and 32 were excluded because they were no longer disease-free. Thus, 391 study subjects participated in the study (i.e., 52.8% of the 740 eligible patients).

On average, respondents were 54.5 years old (± 10.6 years). Most respondents were male, diagnosed with stage I disease, and underwent subtotal gastrectomy. Compared with patients who did not respond to the questionnaire, respondents were more likely to be male, younger, and diagnosed at an earlier stage (Table 1).

**Table 1:** Characteristics of responders and non-responders.

	Responders (N = 391) No (%)*	Non-Responders (N = 349) No (%)*	*p*
Age (years)	54.5 (± 10.6)	57.0 (± 11.6)	< 0.01
Sex			< 0.01
Male	285 (72.9)	216 (61.9)	
Female	106 (27.1)	133 (38.1)	
Time since operation (months)	27.5 (± 3.4)	27.6 (± 3.2)	NS
Stage			< 0.01
Ia	201 (51.7)	162 (46.6)	
Ib	76 (19.5)	58 (16.7)	
II	73 (18.8)	59 (17.0)	
IIIa	30 (7.7)	51 (14.7)	
IIIb	9 (2.3)	18 (5.2)	
Tumor progress			NS
Early	233 (59.6)	158 (40.4)	
Advanced	158 (40.4)	169 (48.4)	
Operation			NS
Subtotal gastrectomy	306 (78.3)	268 (78.6)	
Total gastrectomy	85 (21.7)	74 (21.4)	
Dissection			NS
D1	8 (2.1)	8 (2.3)	
D2	378 (96.7)	336 (96.3)	
Others	5 (1.3)	5 (1.4)	

Abbreviations: NS, not significant; D1, limited lymphadenectomy of the perigastric nodes; D2, extended lymphadenectomy.

* Except for age in years and time since operation, which are expressed as mean ± standard deviation.

### Factors associated with the purchase of private health insurance

In univariate analyses, patients who were younger (OR = 6.39, 95% C.I. 4.00 – 10.18), more educated (OR = 3.08, 95% C.I. = 2.01 – 4.74), religious (OR = 1.57, 95% C.I. = 1.01 – 2.46), employed at the time of diagnosis (OR = 2.85, 95% C.I = 1.81 – 4.46), earning a higher income (OR = 3.54, 95% C.I. = 2.22 – 5.64), and living in an urban area (OR = 2.08, 95% C.I. = 1.27 – 3.40) were more likely to have supplementary PHI at the time of diagnosis. Multivariate analysis showed that younger age (aOR = 5.01, 95% C.I. = 2.05 – 12.24) and higher education (aOR = 2.67, 95% C.I. = 1.04 – 6.86) were independent factors for having PHI (Table 2).

**Table 2:** Determinants of having supplementary private health insurance.

	PHI Status*			
			
	No PHI (N = 190), %	PHI (N = 187), %	Univariate OR(95% C.I.)	Adjusted OR**(95% C.I.)
Age at diagnosis				
> 51 years	154 (67.2)	75 (32.8)		
≤ 50 years	36 (24.3)	112 (75.7)	**6.39 (4.00 – 10.18)**	**5.01 (2.05 – 12.24)**
Martial Status				
Unmarried	25 (55.6)	20 (44.4)		
Married	161 (49.5)	164 (50.5)	1.27 (0.68 – 2.38)	0.51 (0.15–1.71)
Education				
Middle school or below	102 (65.8)	53 (34.2)		
High school or beyond	83 (38.4)	133 (61.6)	**3.08 (2.01 – 4.74)**	**2.67 (1.04 – 6.86)**
Employment at diagnosis				
No	83 (66.4)	42 (33.6)		
Yes	100 (41.0)	144 (59.0)	**2.85 (1.81 – 4.46)**	2.76 (0.91 – 8.33)
Religion				
No religion	65 (58.0)	47 (42.0)		
Having a religion	123 (46.8)	140 (53.2)	**1.57 (1.01 – 2.46)**	2.30 (0.91 – 5.83)
Monthly household income				
< $2,200 USD	88 (66.7)	44 (33.3)		
> $2,200 USD	69 (36.1)	122 (63.9)	**3.54 (2.22 – 5.64)**	2.21 (0.87 – 5.60)
Comorbidity				
Yes	77 (53.4)	67 (46.5)		
No	113 (48.5)	120 (51.5)	1.22 (0.81 – 1.85)	0.65 (0.26 – 1.62)
Smoking at diagnosis				
Yes	80 (47.6)	88 (52.3)		
No	78 (51.7)	73 (48.3)	0.85 (0.55 – 1.32)	1.19 (0.46 – 3.04)
Alcohol at diagnosis				
Yes	101 (49.0)	105 (51.0)		
No	56 (52.8)	50 (47.2)	0.86 (0.54 – 1.37)	1.91 (0.71 – 5.12)
Residential area				
Rural	57 (64.0)	32 (36.0)		
Urban	131 (46.1)	153 (53.9)	**2.08 (1.27 – 3.40)**	2.41 (0.68 – 8.49)

*Among a total of 391 respondents, 14 failed to indicate whether they had private health insurance.

**Standard multivariate logistic regression was performed using all the variables in univariate analysis.

### Impacts of private health insurance on cancer care

Among the 187 subjects who were covered by PHI at the time of diagnosis, nearly all (i.e., 185 subjects) replied that PHI was very helpful or helpful. Only two people stated that PHI was not helpful (Table 3).

**Table 3:** Impact of supplementary private health insurance on various aspects of the cancer care experience.

			Univariate OR(95% C.I.)	Adjusted OR(95% C.I.)
**Access to healthcare**				

	Stage at Diagnosis			
	II – IIIb	Ia – Ib		
			
No PHI	61 (32.3)	128 (67.7)		
Having PHI	48 (25.8)	138 (73.8)	1.37 (0.86 – 2.15)	1.66 (0.93 – 2.99)
	
	Difficulty receiving surgery			
	No	Yes		
			
No PHI	113 (61.4)	71 (38.6)		
Having PHI	115 (62.8)	68 (37.2)	0.94 (0.62 – 1.44)	1.09 (0.59 – 1.74)

**Quality of cancer care**				

	Treatment according to guidelines			
	No	Yes		
			
No PHI	28 (15.2)	156 (84.8)		
Having PHI	16 (8.8)	165 (91.2)	1.85 (0.96 – 3.55)	2.43 (0.98 – 6.04)
	
	Overall satisfaction with care			
	No	Yes		
			
No PHI	28 (15.1)	157 (84.9)		
Having PHI	28 (15.1)	157 (84.9)	1.00 (0.57 – 1.77)	1.21 (0.58 – 2.52)

**Communication & patient autonomy**				

	Involvement in decision making			
	No	Yes		
			
No PHI	71 (38.8)	112 (61.2)		
Having PHI	79 (42.7)	106 (57.3)	0.85 (0.56 – 1.29)	0.77 (0.45 – 1.30)
	
	Reflection of own opinion in decision			
	No	Yes		
			
No PHI	124 (67.8)	59 (32.2)		
Having PHI	121 (66.5)	61 (33.5)	1.06 (0.69 – 1.64)	1.03 (0.59 – 1.79)

**Financial & social independence**				

	Payment of treatment costs by oneself			
	No	Yes		
			
No PHI	109 (57.4)	59 (31.6)		
Having PHI	81 (42.6)	128 (68.4)	**2.92 (1.92 – 4.45)**	**2.07 (1.19 – 3.61)**
	
	Job maintenance after cancer treatment			
	No	Yes		
			
No PHI	29 (29.3)	70 (70.7)		
Having PHI	26 (18.3)	116 (81.7)	**1.85 (1.01 – 3.39)**	0.93 (0.43 – 1.99)

* Adjusted by standard logistic regression for possible confounding variables (e.g., age at diagnosis, educational status, religion, income, residential area, and employment status at the time of diagnosis).

** N = 253, having a job at the time of diagnosis.

#### Access to healthcare services

Having PHI was not significantly associated with earlier stage of disease at diagnosis (aOR = 1.66, 95% C.I = 0.93 – 2.99) or difficulty receiving surgery (aOR = 1.09, 95% C.I = 0.59 – 1.74).

#### Quality of cancer care

Having PHI was not significantly associated with the likelihood of receiving treatment according to KGCS guidelines (aOR = 2.43, 95% C.I = 0.98 – 6.04). In addition, PHI was not associated with overall satisfaction with care (aOR = 1.21, 95% C.I = 0.58 – 2.52).

#### Communication and patient autonomy

Having PHI was not significantly associated with patient involvement in the decision-making process (aOR = 0.77, 95% C.I = 0.45 – 1.30) or reflection of the patient's own opinion in the decision-making process (aOR = 1.03, 95% C.I = 0.59 – 1.79).

#### Financial and social independence

Patients with PHI were more likely to pay their own medical bills, rather than depending on family members (OR = 2.92, 95% C.I. = 1.92 – 4.45). This association remained significant even after adjusting for potential confounding variables (aOR = 2.07, 95% CI 1.19 – 3.61) by standard multiple logistic regression analysis. Among subjects who were employed at the time of cancer diagnosis (i.e., n = 253), those with PHI were more likely to continue working after cancer treatment (OR = 1.85, 95% CI = 1.01 – 3.39). However, this association disappeared after multivariate adjustment (aOR = 0.93, 95% C.I = 0.43 – 1.99).

## Discussion

To our knowledge, this is the first study to examine the impacts of supplementary PHI on cancer care experienced by Korean patients. Our findings reveal that older, less-educated, poorer, and/or unemployed people with cancer are least likely to be covered by PHI. This raises many concerns with regards to inequity, as these groups of people are generally more vulnerable to severe financial burdens when they are affected by serious illnesses such as cancer. These subjects may elect not to purchase PHI because they cannot afford it. Alternatively, these subjects may have been excluded from PHI. In the absence of a well-equipped underwriting system, premiums are usually determined by age and gender, and payments are fixed regardless of the actual medical bills. Thus, there is no financial incentive for the PHI companies to promote PHI to high-risk populations. Indeed, nearly all patients who are over 60 years of age or have pre-existing conditions are excluded from PHI coverage to avoid adverse selections.

Our findings are consistent with a previous Korean study, which found that younger, highly educated, wealthier, and employed patients were most likely to have PHI coverage [21]. Similar findings have been reported in Taiwan, where PHI serves a supplement to mandatory NHI coverage [22]. The Medigap plan, which provides supplementary insurance to Medicare beneficiaries in the US, is more likely to be purchased by elderly patients who are relatively younger, wealthier, better educated and in better health [23]. Taken together, it is unlikely that supplementary PHI functions as an adequate safety net for vulnerable populations.

Interestingly, most cancer survivors expressed appreciation for the assistance provided by supplementary PHI coverage. This result should be interpreted with caution, as these subjects are the winners of "jackpot" insurance. It has been suggested that supplementary PHI is likely to over-insure (and occasionally underinsure) Korean patients. The average benefits awarded to newly diagnosed cancer patients between 2001 and 2005 were approximately $20,000 USD (i.e., 19 million KRW, with an exchange rate of 900 to 1000 during that period) [24], while co-payments during the year of diagnosis are often lower than $10,000 USD. This may explain why many cancer survivors perceive PHI as beneficial, and why they are more likely than other patients to pay their own medical bills.

Our findings indicate that, apart from financial independence, PHI does not significantly impact access to health care, patient autonomy or patient satisfaction. Thus, patients with PHI appear to receive similar treatment as those without PHI. This is in contrast to other studies, which have suggested that PHI improves healthcare accessibility [16,25] and influences quality of care [26].

Our finding that PHI has no significant beneficial influence on cancer care is somewhat disappointing, because previous studies have consistently shown that PHI increases the utilization and expenditure of healthcare in Korea. Those previous data are indicative of insurance effects or moral hazards, at least in outpatient settings [24,27,28]. However, our findings are not surprising when the mechanisms of PHI in Korea are examined more closely. Unlike PHI companies in the US, which provide primary coverage for most of the population and reimburse patients for incurred expenses, PHI companies in Korea do not have access to medical data, and cannot impose regulations on patient care (e.g., with regards to healthcare access, service coverage and quality of care). Thus, our results suggest that expanding PHI coverage will not improve the Korean health care system.

Our study has several limitations. By conducting a retrospective, cross-sectional survey of disease-free survivors of stomach cancer, our results are subject to recall bias and we were unable to assess the patients' experiences during the actual treatment period. Also, patients with advanced disease or recurrence were not included in our analysis, which may have contributed to the relatively high satisfaction rate. Furthermore, a potential selection bias may have resulted from our low response rate (i.e., 52.8%). However, adjustment via the propensity weighting method [29] showed no significant differences from our original findings (data not shown), suggesting that the respondents adequately represented the entire eligible population. Last, as these data were obtained from a general cancer survivorship survey, we were unable to determine specific details regarding patients' PHI coverage, such as the type of plans and the amount of benefits. However, due to the unique characteristics of PHI in Korea, these data were not required for the interpretation of our results.

## Conclusion

Our findings provide valuable lessons for developing countries aiming to achieve universal healthcare coverage in a short time. Policymakers might be easily tempted to promote supplementary PHI to relieve government burdens, assuming PHI will exert a beneficial effect on healthcare performance. However, our findings demonstrate that supplementary PHI neither serves as a safety net for vulnerable patients nor improves cancer care, except for maintaining the financial independence of beneficiaries. Cancer insurance worsened the financial stability of PHI companies, halting sales in 2006 [30], and development of diverse PHI plans has been underway since the Korean government allowed indemnity-type health insurance in 2005. Future studies are needed to determine how new private-public interactions might affect healthcare performance in Korea.

## Competing interests

The authors declare that they have no competing interests.

## Authors' contributions

DWS, KTJ, YHY designed the study. SK, JMB, YWK, KWR, JHL, JHN, STS collected the data. DWS wrote the first draft, which was revised by KTJ and YHY. All authors have read and approved the final manuscript.

## Pre-publication history

The pre-publication history for this paper can be accessed here:



## Supplementary Material

Additional file 1 **Appendix**. Items used in the questionnaire and operational definitions. Click here for file
