# Supplementary material for: Impact of supplementary private health insurance on stomach cancer care in Korea: a cross-sectional study
Source: BMC Health Serv Res. 2009 Jul 31;9:133. doi: 10.1186/1472-6963-9-133 (PMC2726135; doi:10.1186/1472-6963-9-133)
Supplement: Additional file 1 — Appendix. Items used in the questionnaire and operational definitions. [file 1472-6963-9-133-S1.doc]

**Appendix - Items used in the questionnaire and operational definitions.**

| Domain | Question | Problem Responses | Non-Problem Responses |
| --- | --- | --- | --- |
| Access to healthcare | How difficult was it to obtain treatment after you were diagnosed? | Much  Some  A little | None |
| Quality of care | How do you feel about the treatment you received? | Mixed  Mostly Dissatisfied  Unhappy  Terrible | Mostly Satisfied  Delighted  Pleased |
| Communication and patient autonomy | Did you participate in your treatment decisions? | Usually  Sometimes  Never | Always |
|  | How often were your opinions reflected in your treatments? | Usually  Sometimes  Never | Always |
| Financial/social independence | Who paid for most of the cancer treatment costs? | Self  PHI company | Spouse  Children  Parents  Others |
|  | 1) Did you have a job at the time you were diagnosed with gastric cancer?  2) Do you have a job now? | Yes to 1)  Yes to 2) | Yes to 1)  No to 2) |
